# Comparison of affordability and availability of antidepressants in China from 2019 to 2022: evidence from national procurement database

**DOI:** 10.1080/20523211.2026.2719527

**Published:** 2026-10-14

**Authors:** Xiao Han, Qingyuan Xue, Zhao Yang, Jifang Zhou, Bin Jiang

**Affiliations:** a School of Public Health, Shanghai Jiao Tong University, Shanghai, People’s Republic of China; b Gillings School of Global Public Health, University of North Carolina at Chapel Hill, Chapel Hill, NC, USA; c School of Health Management, Inner Mongolia Medical University, Hohhot, People’s Republic of China; d Peking University First Hospital, Peking University, Beijing, People’s Republic of China; e School of International Pharmaceutical Business, China Pharmaceutical University, Nanjing, People’s Republic of China; f Public Policy Research Center, Peking University, Beijing, People’s Republic of China; g School of Pharmaceutical Sciences, Peking University, Beijing, People’s Republic of China

**Keywords:** WHO/HAI, affordability, availability, China, depression, national volume-based procurement

## Abstract

**Background:**

Depression is a widespread mental disorder imposing significant societal burdens. The COVID-19 pandemic exacerbated its prevalence, emphasizing the need for improved access to affordable and effective treatments. This study aims to examine the trends of antidepressant availability and affordability in China from 2019 to 2022 and the role of the National Volume-Based Procurement (NVBP) policy.

**Methods:**

Using nationwide daily procurement data of 19 antidepressants encompassing all medical facilities in China from 2019 to 2022, this study adapted the World Health Organization/Health Action International (WHO/HAI) methodology. Availability was defined as the percentage of health facilities procuring specific antidepressant during the year. Affordability was expressed as the number of days at provincial minimum wage to pay for a one-month course of treatment. Temporal variations were described and explored using fractional logit regression, stratified by originator, NVBP involvement, clinical preference, hospital features, and regions.

**Results:**

Affordability improved steadily between 2019 and 2022 across most antidepressant groups. The largest improvement occurred among first-line NVBP antidepressants, where the monthly expenditure declined by 0.022-day minimum wage annually. In contrast, affordability changed little for first-line non-NVBP medicines (0.015-day decrease per year) and second-line non-NVBP medicines (0.004-day decrease per year). Availability of originator brands declined annually by 0.8 percentage points, while lowest-priced generics increased by 1.1 percentage points. The largest decline occurred in tertiary private facilities, where availability fell by 3.7 percentage points annually. Improvements in availability were mainly observed in secondary and tertiary public facilities, particularly in the East and Northeast regions. Overall, NVBP first-line medicines had 15.5 percentage points higher availability and greater affordability of 0.167-day minimum wage than other antidepressants.

**Conclusion:**

The study highlights the positive influence of the NVBP policy on antidepressant affordability and availability. Regional gaps and facility-specific disparities, though narrowed, still call for policy response.

## 1. Background

Depression is a widespread severe mental disorder carrying significant consequences for both individuals and the society, especially adults aged 60 and above (Institute of Health Metrics and Evaluation, 2023). The COVID-19 pandemic has led to an increase of 53.2 million cases of major depressive disorder and elevating the overall prevalence to 3,152.9 cases per 100,000 persons, estimated to cause 49.4 million disability adjusted life years (DALYs) in 2020 (Santomauro et al., 2021). According to the Lancet World Psychiatric Association Commission (Herrman et al., 2022), despite evidence highlighting depression as a major source of avoidable suffering, it remains a poorly recognised and inadequately understood health condition. Despite various interventions (McCarron et al., 2021), minimally adequate treatment (MAT) rates are only 23% in high-income countries and 3% in low- and lower middle-income countries (Moitra et al., 2022). Ratio of mean cost-of-illness between the depressed and non-depressed is estimated to be 2.58 for direct cost and 2.28 for indirect cost among adults globally (König et al., 2020). The limited availability and high costs of second-line traditional antidepressants significantly impede their use and adherence, especially in low- and middle-income countries (Habte et al., 2023; Todesco et al., 2022). Therefore, it is a global agenda to improve public awareness and ensure the affordable access to depression treatments.

In China, prevalence of depression is an increasingly urgent public health issue for populations of all ages due to a variety of risk factors, which is worsened by the COVID-19 (Ran et al., 2022). Prevalence of major depressive disorder rose from 1,889.0 before the pandemic to 2056.3 in 2020 (per 100,000), and DALYs increased from 378.8 before the pandemic to 412.3 per 100,000 in 2020 (Santomauro et al., 2021). It is estimated that only 9.5% of Chinese patients with depression received any form of treatment, and a mere 0.5% received MAT (Lu et al., 2021). Moreover, regional imbalances have significant implications for patient treatment and recovery (Xiao et al., 2022). Medical resources are disproportionately abundant in provinces with high socioeconomic status, exerting negative spillover effects on the development of health services other provinces. Within each province, proximity to primary healthcare facilities varies (Jia et al., 2022). Last but not least, current utilization of antidepressants is characterised by shortages of medicine with strong clinical evidence and high expenditures (Jia et al., 2022; Yu et al., 2020). Consequently, there is a need for China to enhance the availability and use of first-line antidepressants.

In 2019, the National Healthcare Security Administration (NHSA) initiated the pilot of national volume-based procurement (NVBP) to address the issue of high prices (National Healthcare Security Administration et al., 2019). The NVBP policy takes a volume-for-price strategy by defining the quantity to be purchased before the bid and enabling manufacturers to set prices accordingly (Zhu et al., 2023). Winning manufacturers have to sell the medicine at the bid price, which is typically lower than the market price, for the guaranteed purchase volume. This ensures that manufacturers can predict sales and revenue in advance, reducing financial risk, while the government or procurement body secures the required supply at a controlled cost. By linking price to guaranteed volume, NVBP encourages competitive bidding, lowers drug prices, and ensures stable availability. Generic drugs were required to pass consistency evaluations so that they can be clinically substituted for originator drugs. Health facilities must adhere to the agreed-upon procurement quantities. To date, four of the nine rounds included antidepressants: Escitalopram, Duloxetine, Flupentixol-Melitracen, Fluoxetine, Paroxetine, Sertraline, Venlafaxine, and Citalopram. The NVBP significantly increased volume and reduced prices of antidepressants (Jia et al., 2022; Yuan et al., 2021). Few studies examined the accessibility of antidepressants in the WHO essential drugs list, but these were not representative of current clinical practice (Dong et al., 2023).

This study draws on the survey methodologies employed by the World Health Organization and Health Action International (WHO/HAI) originally developed for essential medicines and adapts it to the analysis of antidepressants under the NVBP policy (Oldfield et al., 2025; World Health Organization & Health Action International, 2008). This study used data from 2019, the period prior to the nationwide implementation of NVBP for antidepressants, to 2022, the most recent year with complete national procurement data after multiple policy rounds. The objective is to comprehensively describe the trends of affordability and availability of antidepressants in China after the NVBP, and variations across different regions and health facilities.

## 2. Methods

### 2.1. Source of data

This retrospective observational study used routinely collected facility-level daily procurement data, following the Reporting of studies Conducted using Observational Routinely-collected health Data (RECORD) guideline. Specifically, we analyzed all daily procurement records from health facilities across mainland China from 2019 (*N* = 16,572) to 2022 (*N* = 22,090; *N* = 10,654 also present in 2019), totaling 28,008 health facilities. All antidepressants were included (*N* = 19). Data were sourced from the Chinese Pharmaceutical Association (CPA), which was responsible for collecting procurement records of health facilities (The Chinese Pharmaceutical Association, 2025).

For each record, we obtained date (daily), buyer, location, drug information (ID, dosage, specification, and conversion factor), unit price, purchased units, and expenditures. Data for the years 2018 and 2023 were excluded for not encompassing all months of the year and thus existing seasonal biases. The year 2020 (*N* = 21,499 facilities) and 2021 (*N* = 22,158) were only used for regression analysis.

Under the zero-mark-up policy, public healthcare facilities are not allowed to add any margin to the procurement prices of drugs when charging patients. As a result, the prices paid by patients are set exactly equal to the procurement prices, ensuring transparency and limiting profit-driven price inflation. This policy makes procurement prices a precise and reliable proxy for the actual drug costs borne by patients, allowing researchers to accurately assess affordability and expenditure at the individual level.

Supplemental Table S1 presents the distribution of health facilities across provinces, categorised by ownership and level. The division of regions was informed by the National Bureau of Statistics of China based on socioeconomic development.

Provincial annual minimum wage standards were obtained from the Ministry of Human Resources and Social Security. Defined daily dose (DDD) data were sourced from WHO Collaborating Centre. International reference prices (IRP) were sourced from the Pharmaceutical Benefits Scheme (PBS) of Australia (Liu et al., 2017; Mu et al., 2022). All expenditures, prices, and minimum wage figures were converted to 2018 USD, accounting for annual inflation rates from the National Bureau of Statistics and exchange rates from the People's Bank of China.

As described in Supplemental Table S2, the data included 19 antidepressants, encompassing selective serotonin reuptake inhibitors (SSRIs), serotonin noradrenaline reuptake inhibitors (SNRIs), tricyclic antidepressants (TCAs), melatonin receptor agonists (MRAs), noradrenergic and specific serotonergic antidepressants (NaSSAs), serotonin receptor antagonist/reuptake inhibitors (SARIs), and noradrenaline reuptake inhibitors (NARIs). According to the national guideline (National Health Commission, 2020) and expert consensus (Chinese Society of Behavioral Medicine & Chinese Society of Behavioral Medicine, 2023; Si et al., 2023), TCAs, known as traditional antidepressants, were classified as secondary treatment options. In contrast, SSRIs, SNRIs, and MRAs were categorised as primary treatments. NARIs and SARIs also fell into the secondary treatment category. Limited clinical evidence was available to support NaSSAs as primary treatments. The National Essential Medicines List (NEML, 2018 edition) featured a range of antidepressants including Amitriptyline, Escitalopram, Doxepin, Fluoxetine, Clomipramine, Paroxetine, and Venlafaxine. Notably, Amitriptyline was the sole antidepressant listed in the WHO/HAI Global Core List (G). In the WHO Model List of Essential Medicines (EML), Amitriptyline and Fluoxetine were listed along with alternative options such as Escitalopram, Fluvoxamine, Paroxetine, Sertraline, and Citalopram.

Because antidepressants were included in the NVBP only after its nationwide implementation (Batch 1), their prices were not affected by the 2019 pilot. Therefore, 2019 was used as the baseline year, and 2022 was selected as the most recent year for assessment of policy impacts.

### 2.2. Variable definition

This study adapted the WHO/HAI methodology for defining key variables and apply them to our dataset covering all medical facilities in China, which is different from the sampling approach proposed by the WHO/HAI (World Health Organization & Health Action International, 2008).

**Classification of Originator and Generic Drugs**: Drug manufacturers were categorised into originator brands (OB), lowest-priced generics (LPGs), and other generic brands. The LPG was determined annually for each health facility.

**Local Drug Pricing Using Defined Daily Dose Cost (DDDc)**: In contrast to the WHO/HAI's median unit prices (MUPs), the study employed the defined daily doses (DDDs) price of each drug to represent the average pricing, i.e. DDDc. The DDDc was calculated as total aggregated expenditure divided by total aggregated DDDs, not on a record-by-record basis, indicating the average individual cost of suggested daily usage. Compared with MUPs, DDDc is a comparable price between antidepressants.

**International Price Comparison**: To facilitate cross-country comparison of prices, we used median price ratio (MPR), defined by the WHO/HAI as ratio of median medicine unit price to the IRP.

$$\mathrm{MPR}=\frac{\mathrm{Median}\;\mathrm{local}\;\mathrm{unit}\;\mathrm{price}}{\mathrm{International}\;\mathrm{reference}\;\mathrm{unit}\;\mathrm{price}}$$

**Availability**: Following the WHO/HAI methodology, availability was defined as the percentage of health facilities procuring specific antidepressant. Therefore, availability was constructed separately for each antidepressant type and survey year, stratified by facility features if applicable. Availability measures the presence of a drug across health facilities, with high availability indicating that more health facilities are equipped with the drug at the time of survey. In our study, if a specific medicine was procured at least once during the year, it was considered an available medicine at the facility. In subgroup analysis, to obtain aggregated features of different antidepressant categories, antidepressant-specific availabilities were pooled with averages. Given facility feature A (optional), antidepressant name i, and the survey year t, availability could be defined as:

$$\mathrm{Availabilit}y_{A,i,t}=\frac{\mathrm{Number}\;\mathrm{of}\;\mathrm{facilities}\;\mathrm{with}\;\mathrm{feature}\;A\;\mathrm{where}\;\mathrm{antidepressant}\;i\;\mathrm{was}\;\mathrm{available}\;\mathrm{in}\;\mathrm{year}\;t}{\mathrm{Number}\;\mathrm{of}\;\mathrm{all}\;\mathrm{facilities}\;\mathrm{with}\;\mathrm{feature}\;A\;\mathrm{in}\;\mathrm{year}\;t}\times 100\text{\%}$$

**Affordability**: Affordability is the cost of a defined course of treatment (30 days for chronic conditions) in relation to the average income or minimum wage of the local population, which is specific to each antidepressant and facility (Oldfield et al., 2025; World Health Organization & Health Action International, 2008). In our study, affordability for each antidepressant was calculated on the facility level, defined as monthly expenditure (30 multiplied by DDDc, the cost per defined daily dose), divided by the provincial minimum wage. An affordability less than one indicated an affordable price, indicating that the cost of a 30-day treatment is less than one day of minimum wage. In subgroup analysis, to obtain aggregated features of different antidepressant and facility categories, antidepressant-specific and facility-specific affordabilities were also pooled with averages. Given facility name k, antidepressant name i, and the survey year t, mean DDDc was first calculated if multiple records were present, and availability could be then defined as:

$$\mathrm{Affordabilit}y_{k,i,t}=\frac{30\;*\;\mathrm{mean}\;\mathrm{DDDc}\;\mathrm{of}\;\mathrm{antidepressant}\;i\;\mathrm{in}\;\mathrm{facility}\;k\;\mathrm{in}\;\mathrm{year}\;t}{\mathrm{Minimum}\;\mathrm{wage}\;\mathrm{in}\;\mathrm{year}\;t\;\mathrm{for}\;\mathrm{the}\;\mathrm{province}\;\mathrm{where}\;\mathrm{facility}\;k\;\mathrm{is}\;\mathrm{located}}$$

### 2.3. Statistical analyses

Availability, affordability, and MPR of antidepressants were described, stratified by categories including drug features, facility types, and regions. Growth rates between 2022 and 2019 were calculated.

To assess variations in temporal trends by subgroups, data were aggregated on the antidepressant level on a yearly basis, stratified by facility features if applicable. Given that both affordability and availability can be interpreted as bounded proportions (0–1) and may have complex distributions, we then performed fractional logistic regression with robust standard error on aggregated data and reported average marginal effect (AME) instead of the beta coefficient. Given our continuous outcome variable, the AME of the predictor year estimates the average annual change in the predicted value of the outcome, holding all other variables at their observed values. This provides a direct interpretation of the effect size in the original units of the outcome variable. Subgroups included: (1) manufacturers (OB, LPGs, or others); (2) guideline-based preferences (first-line, second-line, or others) and policy-based preferences (included in the NVBP list or not); (3) level (primary, secondary, or tertiary) and ownership (public or private) of health facilities; (4) region of health facilities (east, middle, northeast, or west) as categorised in Supplemental Table S1.

Subgroup-specific availability and affordability for each antidepressant were calculated using the same regression model, setting year as the variable of concern and further controlling for fixed effects of specific antidepressants to account for time-invariant characteristics of each medicine. The AMEs of year were visualised using forest plots along with 95% confidence intervals (CIs). Similarly, comparison between categories were performed by setting the categorical variables as the explanatory variable of concern, controlling for year and antidepressant fixed effects if applicable.

Significance levels were set at *P* < 0.001***, *P* < 0.01**, and *P* < 0.05*, with 95% CIs. Descriptions and forest plots were performed in Python 3.11.5, and regressions were run in Stata MP 17.

## 3. Results

### 3.1. Description of included antidepressants

Supplemental Table S3 presents the competition landscape of each drug from 2019 to 2022. The original manufacturers of seven antidepressants were absent from the procurement records. The original manufacturers of Agomelatine and Trazodone entered the market later than generic manufacturers. For six antidepressants, only one to two generic manufacturers existed. The number of generic manufacturers for Escitalopram increased from 7 in 2019–13 in 2022, while Sertraline and Venlafaxine demonstrated a higher degree of competition with over 10 or manufacturers.

### 3.2. Temporal patterns of antidepressants

Table 1 presents affordability and availability of antidepressants grouped by mechanisms of action in 2019 and 2022. Affordability and availability varied markedly across antidepressant classes. All antidepressants had affordability values below one, indicating overall affordability. Affordability improved for most drugs, except for the TCAs amitriptyline (0.01–0.01, 5.1%) and doxepin (0.01–0.01, 27.1%).

**Table 1.** Affordability and availability of antidepressants, categorised by mechanism of action.

	Affordability			Availability		
	2019	2022	Change	2019	2022	Change
**SSRIs**						
Citalopram	0.08	0.04	−55.1%	10.9%	11.0%	1.1%
Escitalopram	0.13	0.06	−55.0%	29.8%	36.2%	21.7%
Fluoxetine	0.06	0.01	−75.3%	15.8%	19.0%	20.3%
Fluvoxamine	0.08	0.07	−11.8%	10.1%	12.8%	27.6%
Paroxetine	0.06	0.03	−55.6%	33.8%	34.1%	1.0%
Sertraline	0.06	0.03	−56.6%	33.5%	31.4%	−6.3%
**SNRIs**						
Duloxetine	0.20	0.04	−78.5%	17.3%	24.6%	41.9%
Milnacipran	0.51	0.45	−12.8%	3.1%	1.9%	−38.7%
Venlafaxine	0.12	0.06	−51.4%	22.5%	25.1%	11.3%
**TCAs**						
Amitriptyline	0.01	0.01	5.1%	12.7%	7.6%	−40.1%
Clomipramine	0.02	0.02	−21.9%	7.4%	6.2%	−16.7%
Doxepin	0.01	0.01	27.1%	16.9%	13.8%	−18.4%
Maprotiline	0.03	0.03	−6.3%	0.8%	0.5%	−34.4%
Mianserin	0.13	0.12	−10.5%	2.1%	3.1%	48.5%
**Others**						
MRAs: Agomelatine	0.21	0.17	−17.3%	6.6%	11.5%	74.6%
NaSSAs: Mirtazapine	0.11	0.11	−2.1%	16.7%	22.1%	31.9%
NARIs: Reboxetine	0.09	0.06	−32.9%	0.5%	0.1%	−78.3%
SARIs: Trazodone	0.25	0.22	−11.2%	17.1%	19.2%	12.7%
Combination: Flupentixol-Melitracen	0.04	0.03	−20.3%	60.0%	60.8%	1.4%

Notes: Affordability < 1 indicates that the cost of a 30-day treatment is less than one day of minimum wage. Availability is operationalised as the percentage of health facilities that supplied specific antidepressants during the survey year. SSRI, selective serotonin reuptake inhibitor; SNRI, serotonin noradrenaline reuptake inhibitor; TCA, tricyclic antidepressant; MRA, melatonin receptor agonist; NaSSA, noradrenergic and specific serotonergic antidepressant; NARI, noradrenaline reuptake inhibitor; SARI, serotonin receptor antagonist/reuptake inhibitor.

Flupentixol–melitracen showed the highest availability (59.9%−60.8%, 1.4%), followed by the SSRIs escitalopram (29.8%−36.2%, 21.7%), paroxetine (33.8%−34.1%, 1.0%), and sertraline (33.5%−31.4%, −6.3%). The lowest availability was observed for reboxetine (NARIs, 0.5%−0.1%, −78.3%), maprotiline (TCAs, 0.8%−0.5%, −34.4%), and mianserin (TCAs, 2.1%−3.1%, 48.5%). Agomelatine (MRAs) experienced the fastest growth in availability (6.6%−11.5%, 74.6%).

With the exception of sertraline (33.5%−31.4%, −6.3%) and milnacipran (3.1%−1.9%, −38.7%), the availability of SSRIs and SNRIs increased to varying degrees (1.0%−41.9%). In contrast, except for mianserin (2.1%−3.1%, 48.5%), TCAs generally showed declines in availability (16.7%−40.1%).

Table 2 provides affordability and availability of antidepressant categories in 2019 and 2022. In comparison to OBs (0.08-0.06, −21.2%) and other generics (0.10-0.06, −39.2%), LPGs had the lowest affordability and experienced the largest decline (0.08-0.04, −48.5%). In contrast to OBs (28.6%−27.8%, −2.7%) and other generics (4.8%−4.6%, −4.8%), the availability of LPGs increased markedly (17.5%−22.6%, 28.7%), although it remained lower than that of OBs.

**Table 2.** Affordability and availability of antidepressants, by categories.

	Affordability			Availability		
	2019	2022	Change	2019	2022	Change
**LPG**						
LPG	0.08	0.04	−48.5%	17.5%	22.6%	28.7%
OB	0.08	0.06	−21.2%	28.6%	27.8%	−2.7%
Others	0.10	0.06	−39.2%	4.8%	4.6%	−4.8%
**List**						
NEML: NEML	0.09	0.04	−54.3%	25.7%	28.6%	10.9%
NEML: Non-NEML	0.08	0.05	−31.1%	34.6%	33.9%	−2.1%
G: G	0.01	0.01	5.1%	12.7%	7.6%	−40.1%
G: Non-G	0.08	0.05	−42.1%	31.5%	32.2%	2.2%
EML: EML	0.05	0.01	−71.4%	14.9%	16.7%	11.8%
EML: A	0.08	0.04	−48.8%	29.0%	30.5%	4.9%
EML: Non-EML	0.09	0.06	−28.7%	34.5%	34.4%	−0.1%
**NVBP**						
NVBP: NVBP	0.08	0.04	−53.1%	36.3%	37.3%	2.6%
NVBP: Non-NVBP	0.11	0.11	0.7%	13.4%	16.1%	19.9%
**Region**						
East	0.08	0.05	−36.6%	33.9%	36.5%	7.8%
Middle	0.08	0.05	−36.4%	29.9%	32.4%	8.4%
West	0.09	0.04	−57.3%	30.3%	25.6%	−15.6%
Northeast	0.10	0.07	−37.0%	23.8%	29.2%	22.6%
**Level**						
Primary	0.06	0.04	−30.9%	29.4%	27.0%	−8.2%
Secondary	0.07	0.04	−40.4%	37.1%	39.8%	7.4%
Tertiary	0.09	0.05	−42.3%	48.4%	53.1%	9.6%
**Ownership**						
Private	0.07	0.04	−37.9%	33.6%	30.5%	−9.3%
Public	0.08	0.05	−41.9%	31.2%	32.1%	2.8%

Notes: Affordability < 1 indicates that the cost of a 30-day treatment is less than one day of minimum wage. Availability is operationalised as the percentage of health facilities that supplied specific antidepressants during the survey year. LPG, lowest-priced generics; OB, originator brand; NEML, National Essential Medicines List; G, WHO/HAI Global Core List; EML, WHO Model List of Essential Medicines; NVBP, National Volume-Based Procurement.

The affordability of NEML drugs declined substantially (0.09-0.04, −54.3%), shifting from higher than that of non-NEML drugs to lower. Although the availability of NEML drugs increased slightly (25.7%−28.6%, 10.9%), it remained below that of non-NEML drugs, whose availability was largely stable (34.6%−33.9%, −2.1%).

In contrast to group G (amitriptyline), non-G drugs showed improved affordability (0.08-0.05, −42.1%) and increased availability (31.5%−32.2%, +2.2%).

Affordability decreased across EMLs (0.05-0.01, −71.4%), As (0.08-0.04, −48.8%), and non-EMLs (0.09-0.06, −28.7%), with non-EMLs consistently exhibiting the lowest affordability. Availability for non-EMLs declined marginally (34.5%−34.4%, −0.1%) but remained higher than the increasing availability of EMLs (14.9%−16.7%, 11.8%) and As (29.1%−30.5%, 4.9%).

While affordability of non-NVBP antidepressants remained stable (0.11-0.11, 0.7%), NVBP antidepressants had better affordability that also improved substantially (0.08-0.04, −53.1%). Availability of NVBP antidepressants (36.4%−37.3%, 2.6%) was consistently higher than that of non-NVBP antidepressants (13.4%−16.1%, 19.9%).

Affordability in the west region improved the most (0.09-0.04, −57.3%), but affordability in the northeast remained the highest (0.10-0.07, −37.0%). Increase of availability in the northeast region was the highest (23.8%−29.2%, 22.6%). However, availability in the west region decreased (30.3%−25.6%, −15.6%).

Although affordability was negatively associated with higher tiers of health facilities, higher-level facilities showed greater improvement. Primary health facilities had the lowest availability and experienced a decline (29.4%−27.0%, −8.2%), whereas secondary (37.1%−39.8%, 7.4%) and tertiary (48.4%−53.1%, 9.6%) facilities showed notable increases in availability.

Compared with private health facilities (0.07-0.04, −37.9%), public facilities had worse affordability but exhibited a larger improvement (0.08-0.05, −41.9%). Availability decreased in private facilities (33.6%−30.5%, −9.3%) but increased in public facilities (31.2%−32.1%, 2.8%).

Figure 1 displays the annual change in affordability, expressed as the AME, across different antidepressant and facility categories. Overall, affordability improved steadily from 2019 to 2022 across most brands, health facilities, and regions, with the exception of the West region. The largest improvement was associated with first-line NVBP antidepressants, for which affordability decreased by 0.022 days of minimum wage per year (95% CI [−0.035, −0.009], *P* < 0.001), indicating that the number of days of minimum wage required to cover monthly treatment costs declined annually in this group. In comparison, the changes for first-line non-NVBP antidepressants were smaller, corresponding to an annual decrease of 0.015 days (95% CI [−0.105, 0.075], *P* = 0.751), while second-line non-NVBP antidepressants showed only a minor annual decrease of 0.004 days (95% CI [−0.027, 0.019], *P* = 0.750). Together, these patterns indicate that improvements in affordability over the study period were primarily driven by first-line antidepressants included in the NVBP program.

**Figure 1.**
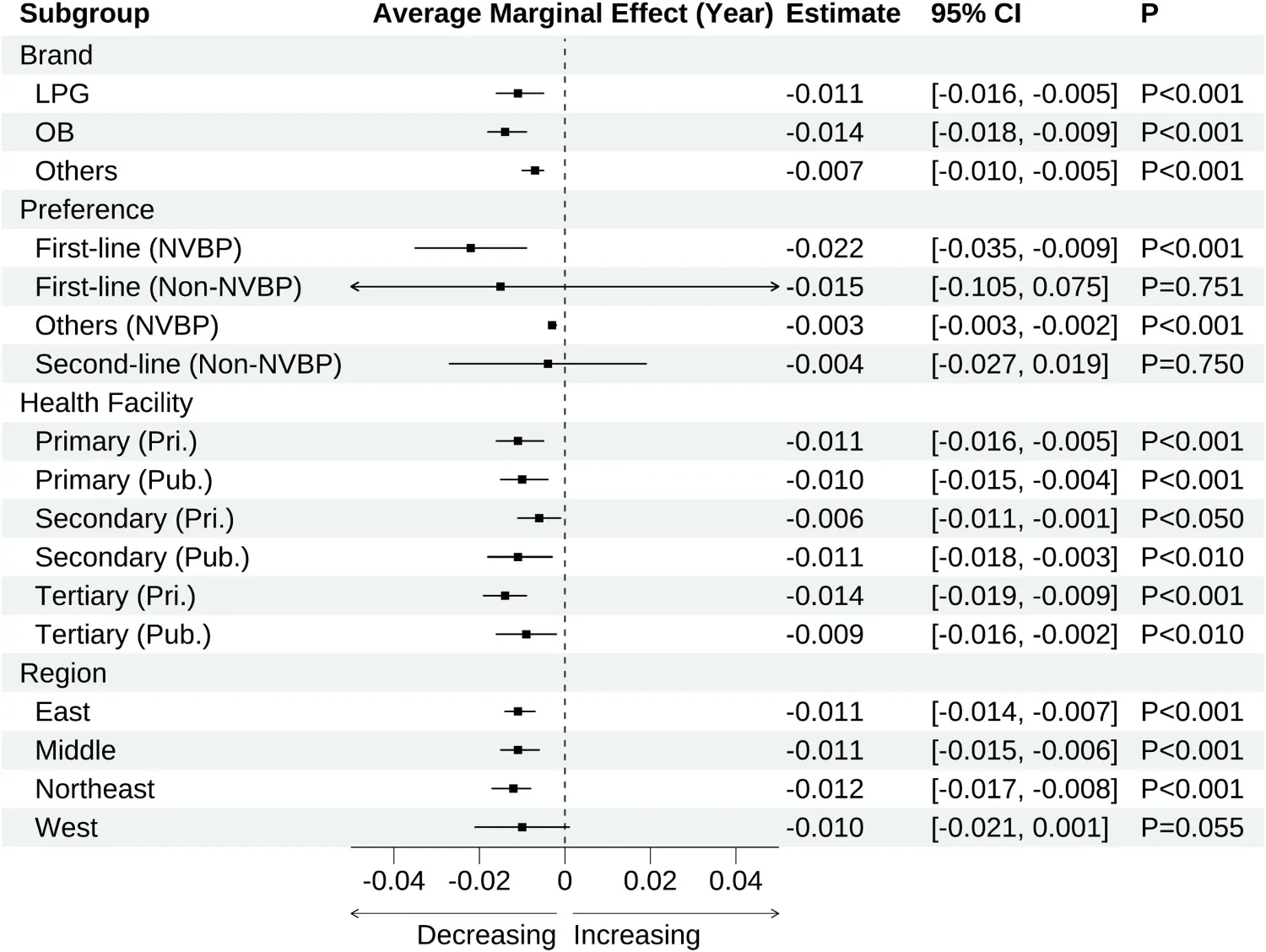
Annual Changes in Affordability by Brand, Guideline First-line Recommendation and NVBP Status, Health Facilities, and Regions. Affordability < 1 indicates that the cost of a 30-day treatment is less than one day of minimum wage. Subgroup analysis of the temporal trends was performed using fractional logit regression with robust standard errors, controlling for fixed effects of specific antidepressants. The average marginal effect (AME) represents the average annual change in the predicted value of affordability. The AMEs, 95% confidence intervals, and *P* values of all subgroup models were visualised in the forest plot. LPG, lowest-priced generics; OB, originator brand; NVBP, National Volume-Based Procurement; Pub., public; Pri., private.

Figure 2 displays the annual change in predicted availability (AME) across antidepressant and facility categories from 2019 to 2022. Opposite trends were observed across categories. Availability of OBs declined by an estimated 0.8 percentage points per year (95% CI [−0.012, −0.004], *P* < 0.001), indicating a gradual reduction in the proportion of facilities procuring these products. A larger decline occurred in tertiary private health facilities, where predicted availability decreased by 3.7 percentage points annually (95% CI [−0.051, −0.022], *P* < 0.001), representing the most substantial reduction among facility types. In contrast, the availability of LPGs increased by 1.1 percentage points per year (95% CI [0.006, 0.015], *P* < 0.001), reflecting a steady expansion in facility-level procurement. This improvement was particularly evident in public health facilities located in the East and Northeast regions, both coastal and relatively more economically developed areas.

**Figure 2.**
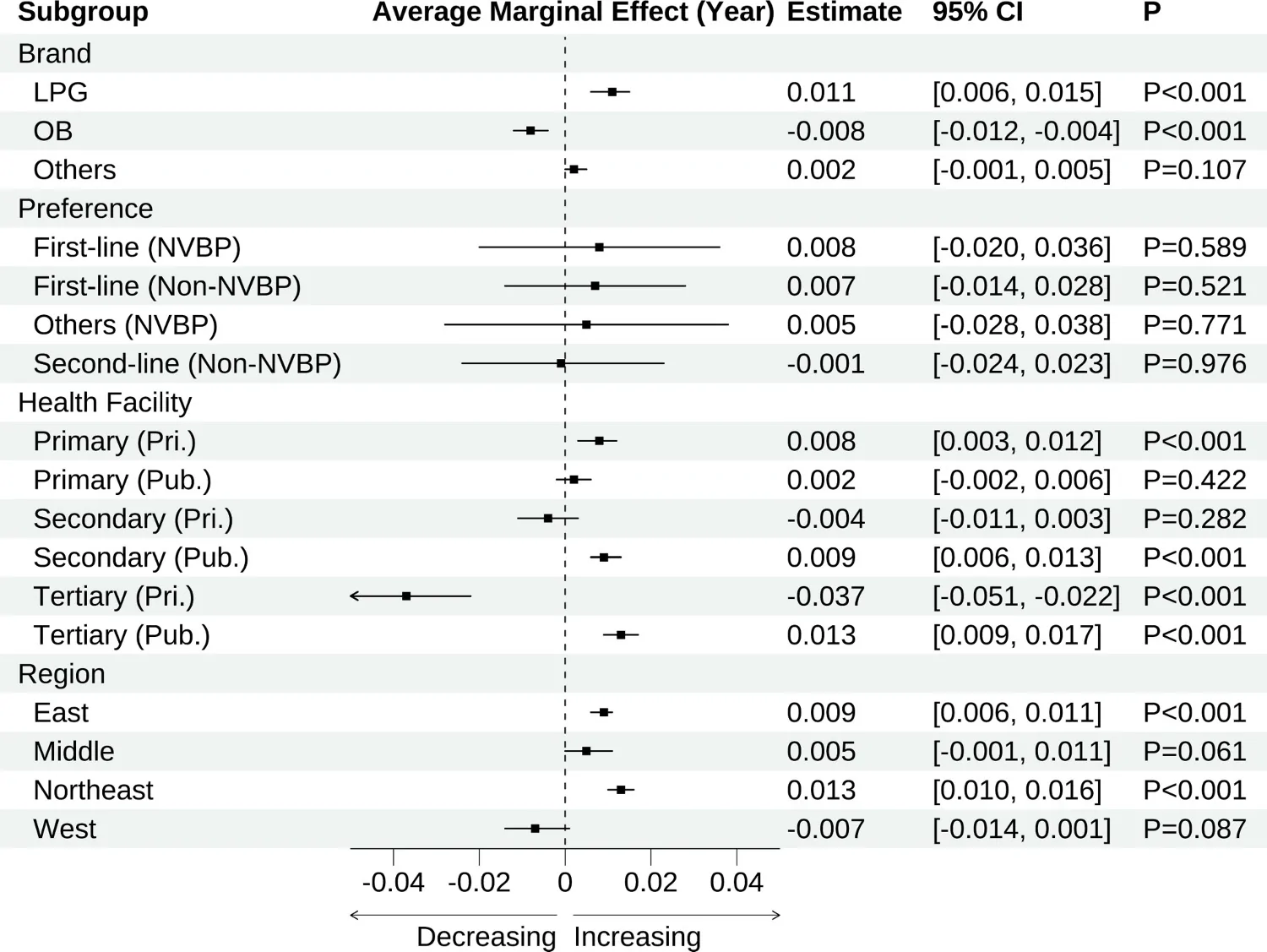
Annual Changes in Availability by Brand, Guideline First-line Recommendation and NVBP Status, Health Facilities, and Regions. Availability is operationalised as the percentage of health facilities that supplied specific antidepressants during the survey year. Subgroup analysis of the temporal trends was performed using fractional logit regression with robust standard errors, controlling for fixed effects of specific antidepressants. The average marginal effect (AME) represents the average annual change in the predicted value of availability. The AMEs, 95% confidence intervals, and *P* values of all subgroup models were visualised in the forest plot. LPG, lowest-priced generics; OB, originator brand; NVBP, National Volume-Based Procurement; Pub., public; Pri., private.

Table 3 presents the average subgroup differences in availability and affordability from 2019 to 2022, controlling for antidepressants and year. Overall, the results highlight differences in availability and affordability across medicine and facility characteristics.

**Table 3.** Comparison of availability and affordability, by categories.

	Availability		Affordability	
	AME	95% CI	AME	95% CI
LPG	Ref.		Ref.	
OB	−0.075***	[−0.101, −0.049]	0.061***	[0.048, 0.073]
Other Generic	−0.134***	[−0.150, −0.118]	0.015***	[0.007, 0.024]
First-line (NVBP)	Ref.		Ref.	
First-line (Non-NVBP)	−0.173***	[−0.210, −0.135]	0.167***	[0.076, 0.258]
Second-line (Non-NVBP)	−0.155***	[−0.194, −0.116]	0.018	[−0.012, 0.049]
Tertiary (Pub.)	Ref.		Ref.	
Primary (Pri.)	−0.231***	[−0.246, −0.215]	−0.009*	[−0.018, −0.001]
Primary (Pub.)	−0.236***	[−0.248, −0.224]	−0.024***	[−0.034, −0.013]
Secondary (Pri.)	−0.196***	[−0.209, −0.184]	−0.014**	[−0.023, −0.005]
Secondary (Pub.)	−0.124***	[−0.135, −0.113]	−0.015*	[−0.026, −0.003]
Tertiary (Pri.)	−0.153***	[−0.177, −0.130]	0.003	[−0.006, 0.012]
East	Ref.		Ref.	
Middle	−0.015*	[−0.029, −0.001]	0.010**	[0.003, 0.018]
Northeast	−0.029**	[−0.046, −0.011]	0.015***	[0.007, 0.022]
West	−0.027***	[−0.043, −0.012]	0.004	[−0.012, 0.019]

Notes: Affordability < 1 indicates that the cost of a 30-day treatment is less than one day of minimum wage. Availability is operationalised as the percentage of health facilities that supplied specific antidepressants during the survey year. Fractional logit regression with robust standard errors was used to estimate the average annual change in the predicted value of the outcome (displayed as AMEs). AME, average marginal effect; LPG, lowest-priced generics; OB, originator brand; NVBP, National Volume-Based Procurement; Pub., public; Pri., private.

For availability, antidepressants classified as LPGs had 7.5 percentage points higher availability than other medicines (95% CI [−0.101, −0.049], *P* < 0.001). NVBP medicines receiving first-line guideline recommendations showed an even larger increase of 15.5 percentage points (95% CI [−0.194, −0.116], *P* < 0.001). Availability was 12.4 percentage points higher in tertiary health facilities (95% CI [−0.135, −0.113], *P* < 0.001) and only 1.5 percentage points higher in facilities located in the East region (95% CI [−0.029, −0.001], *P* < 0.05).

For affordability, the pattern differed. Antidepressants classified as LPGs were less affordable by 0.015 days of minimum wage (95% CI [0.007, 0.024], *P* < 0.001). In contrast, NVBP medicines with first-line guideline recommendations were more affordable by 0.167 days of minimum wage (95% CI [0.076, 0.258], *P* < 0.001) than other first-line antidepressants. Facilities located in the East region also showed a modest improvement in affordability (AME = 0.010, 95% CI [0.003, 0.018], *P* < 0.01). In contrast, antidepressants in tertiary public health facilities were slightly less affordable than others, with differences ranging from 0.009–0.024 days of minimum wage.

### 3.3. Antidepressants stratified by regions

Supplemental Table S4 presents the regional differences in availability and MPR of antidepressants in 2019 and 2022, grouped by manufacturer, treatment preference, and participation in the NVBP policy. First-line NVBP medicines, especially LPGs, had the highest availability and the lowest MPR across regions. Both availability and MPR declined after the NVBP policy. Only LPGs of first-line NVBP antidepressants had a MPR lower than one (range 0.52-0.93), indicating prices below international levels. OB availability dropped sharply (range −21.9% to −40.8%) due to substitution by LPGs, except in the northeast. Availability and MPR of LPGs improved most in the middle (availability 20.6%; MPR −56.0%) and west (availability 13.4%; MPR −72.9%) region.

First-line non-NVBP antidepressants also showed improved availability and MPR. In contrast to first-line NVBP, there was no evidence of the substitution between LPGs and OBs except in the east region (LPGs: 34.2%; OBs: −7.7%). LPG MPR declined (−30.5% to −3.5%) while OB MPR increased (11.6%−74.7%).

LPGs of second-line non-NVBP maintained relatively high availability but declined in the middle (−19.7%) and west (−35.0%) regions. However, LPG MPR was rising in all regions (13.2%−48.8%), raising costs for patients on second-line treatments.

### 3.4. Antidepressants stratified by features of health facilities

Supplemental Table S5 provides the differences in availability and MPR of antidepressants in 2019 and 2022, grouped by manufacturer and treatment preference, and participation in the NVBP policy, further stratified by level of health facilities.

Availability of treatments increased with the level of health facility. In 2022, first-line NVBP LPGs were less available at primary facilities (public 50.3%; private 55.8%) than at secondary (public 73.9%; private 75.0%) and tertiary (public 88.2%; private 88.6%) facilities. First-line non-NVBP antidepressants, both LPGs and OBs, were generally poorly available (maximum 8.9%), except for secondary (LPGs 20.6%) and tertiary (LPGs 40.8%) public facilities. Second-line non-NVBP antidepressants were widely available across all facility levels (16.7%−70.6%).

Public and private health facilities showed contrasting trends across all levels. Primary public facilities reduced second-line non-NVBP antidepressant availability (31.2%−26.3%, −15.7%), while primary private facilities increased it (20.0%−22.3%, 11.2%). This pattern reversed at secondary (public 50.8%−51.2%, 0.9%; private 45.0%−25.9%, −42.5%) and tertiary levels (public 69.0%−70.6%, 2.2%; private 53.6%−16.7%, −68.9%). For first-line non-NVBP LPGs, MPR changes ranged from −30.5% to 52.7%, with the only increases and largest decrease (tertiary) occurring in private facilities. Tertiary private facilities also saw sharp declines in availability for LPGs (28.6%−8.3%, −70.8%) and OBs (3.6%−2.3%, −36.4%), despite increases in other facility categories.

## 4. Discussions

### 4.1. Major findings on affordability and availability

This study identified significant changes in the affordability and availability of antidepressants between 2019 and 2022. Overall, antidepressant prices remained within an acceptable range for patients, and affordability showed universal improvement, particularly for NVBP-listed medications. The availability of first-line NVBP antidepressants increased and remained relatively high over the study period, whereas second-line antidepressants, mainly TCAs, showed a modest decline in availability but continued to be supplied at a baseline level.

These trends may reflect the influence of the NVBP policy. The NVBP policy progressively included various first-line antidepressants and negotiated substantial price reductions from manufacturers by ensuring mandatory volumes procured in health facilities. Our results suggest a marked improvement in the affordability of NVBP antidepressants compared with non-NVBP ones, which may indicate that price reductions under the NVBP policy were associated with increased patient access. Additionally, NVBP antidepressants tended to be more available than non-NVBP drugs, which may suggest that the policy prioritised commonly prescribed first-line antidepressants. The guaranteed procurement volumes under the NVBP policy reduced the likelihood that these drugs would be excluded from health facilities, thereby helping to maintain a stable supply. The policy may also have been associated with changes in traditional antidepressants such as TCAs, which coincided with a slight reduction in their availability. However, the essential medicine policy required facilities to maintain a minimum supply of these non-NVBP essential drugs. Notably, first-line treatments such as MRAs (e.g. Agomelatine) were supplied by a limited number of manufacturers, resulting in low market competition for inclusion in the NVBP. This limited competition might help explain their lower availability despite recommendations as first-line antidepressants.

The affordability of LPG antidepressants was the highest among all categories examined and showed notable improvement between 2019 and 2022. Their availability also increased, although it remained lower than that of OBs. The observed patterns suggest that LPGs may have partially substituted for OBs across multiple regions and types of health facilities. These trends appear to align with the introduction of the NVBP policy (Wang et al., 2022). Bid-winning manufacturers under the NVBP were predominantly generic, and the policy encourages these generics as the primary, cost-effective options in health facilities, potentially displacing non-winning generics and OBs. The mandatory implementation of NVBP medications across health facilities served to promote wider availability of winning antidepressants. Taken together, these findings suggest that the NVBP policy may have played a crucial role in improving patient access to affordable, high-quality antidepressants through generic substitution. Nevertheless, OBs continued to retain a notable market share, which reflect their established clinical reputation and patient or provider preference. Future policies need to monitor the market position of OBs and the preferences of patients and physicians to safeguard the diversity, affordability, and consistent availability of antidepressants.

Our findings also indicate disparities in affordability and availability of antidepressants across the NEML, G, and EML. First, antidepressants listed in the NEML were more affordable than those outside the list, although their availability remained comparatively low. This pattern may reflect the combined influence of both the regulatory framework and data characteristics. For one thing, medicines outside the list can be priced freely and facilities therefore had the economic incentives to maintain their supply. For another, four NVBP antidepressants were listed and three were not, thus observed differences partly reflect differences between these NVBP antidepressants. Second, for antidepressants outside the G list, both affordability and availability showed improvement, while Amitriptyline, the only G-listed antidepressant, displayed a declining trend. One possible explanation is that antidepressants outside the G list included all NVBP antidepressants, whose affordability and availability were likely influenced by NVBP pricing and procurement policies. Third, both affordability and availability of antidepressants within and outside the EML improved during the study period. Antidepressants outside the EML exhibited the worst affordability but the highest availability. This pattern is similar to that observed for the NEML and may be related to the substantial overlap between the two lists.

While the present study demonstrates improvements in the affordability and availability of antidepressants following policy implementation, these supply-side changes do not necessarily translate into improved treatment coverage for depression. Access to effective treatment is also shaped by several demand-side and system-level factors. In many settings, substantial diagnosis gaps persist, with a large proportion of individuals with depression remaining undiagnosed or untreated (Lu et al., 2021; Moitra et al., 2022; Ran et al., 2022). Stigma surrounding mental illness may discourage help-seeking and reduce treatment adherence (Du et al., 2023), while shortages of mental health professionals and the uneven distribution of mental health services may further limit access to care (World Health Organization, 2025; Xiao et al., 2022). Consequently, although policies that improve medicine affordability and availability represent an important step toward strengthening mental health treatment systems, additional efforts aimed at improving detection, reducing stigma, and expanding service capacity are also necessary to ensure that patients with depression can effectively receive and benefit from available medications.

### 4.2. Equity across regions and facilities

The study highlights regional variations in the affordability and availability of antidepressants. Across all regions, first-line NVBP antidepressants consistently showed the highest availability, reflecting a rational medication structure. Health facilities appear to follow clinical guidelines and international best practices in their selection of antidepressants, which could contribute to improved patient outcomes. In regions with lower economic and healthcare capacity (central and western areas), the affordability and availability of first-line antidepressants improved substantially, which may indicate a narrowing of regional disparities and a potentially more equitable distribution of healthcare resources. However, availability of second-line NVBP LPGs remained relatively high and their MPRs showed an increasing trend, indicating that further improvements may still be needed. In some provinces, inadequate healthcare resources may remain a challenge for patients seeking affordable access to medications. Governments and healthcare authorities could consider further measures to increase healthcare resource allocation in these areas.

Across the three tiers of health facilities, the availability of first-line antidepressants has generally increased, with higher-tier facilities demonstrating better availability, though notable disparities persist. Higher-tier facilities typically have greater medical resources and more robust supply chains, enabling them to offer a wider range of antidepressants and partly explaining why patients seek care at these facilities. These findings suggest that it remains important to ensure that lower-tier facilities continue to provide access to essential antidepressants. In the private healthcare sector, antidepressant availability varied compared with public facilities, potentially reflecting the tendency of private decision-makers to offer a broader selection to attract patients. Future studies could further examine the incentives influencing antidepressant selection in both public and private health facilities in China.

Across all facility levels and ownership types, second-line antidepressants, primarily traditional TCAs, were widely available. However, their availability has grown more slowly or even declined from 2019 to 2022, which may suggest a gradual replacement of traditional antidepressants with newer-generation alternatives. This trend likely reflects increasing acceptance of newer-generation antidepressants within the medical community, as both patients and clinicians may perceive them to be more effective and safer. Nevertheless, it may still be important to maintain access to traditional antidepressants to meet individualised treatment needs.

### 4.3. Limitations

There are several limitations to this study. First, we utilised procurement data of health facilities, which prevented us from accounting for variations between outpatient and inpatient services. The absence of reimbursement data, such as out-of-pocket proportion, renders us unable to accurately assess the true economic burden of depression.

Second, the data only included procurement prices. For one thing, although patient prices equalled procurement prices under the zero-mark-up policy, data on additional costs incurred by healthcare facilities, such as dispensing fees, storage expenses, inventory management, and other operational overheads, were not available. These additional costs could contribute to the overall economic burden of medications, but their absence in our data limits the ability to fully capture the total cost of drug provision and cost components. For another, improved availability and affordability of medicines do not automatically translate into increased treatment coverage, as barriers such as diagnostic gaps, stigma, limited provider capacity, and uneven distribution of mental health services may persist.

Third, the study period coincided with the COVID-19 pandemic. Pandemic-related factors, such as disruptions to pharmaceutical manufacturing and logistics, transportation restrictions, reduced outpatient visits, and shifts in healthcare service delivery, may have affected both medication supply and patient access to care (Tirivangani et al., 2021; World Health Organization, 2020). Changes in healthcare-seeking behaviour, including postponed consultations or interrupted treatment due to lockdowns or infection concerns, may also have influenced medication demand (Baatiema et al., 2025; Clement et al., 2021). Because these contextual factors cannot be separated from policy effects in our analysis, the observed procurement changes should be interpreted with caution. Nevertheless, despite these potential disruptions, the observed improvements in medicine affordability and availability highlight for future studies the importance of ensuring that patients with depression are effectively diagnosed and able to access and receive appropriate treatment.

## 5. Conclusion

This study analyzed nationwide procurement data of 19 antidepressants from 2019 to 2022, using an adapted WHO/HAI methodology to evaluate affordability and availability. Under an observational design, the results showed a marked improvement in the affordability of all antidepressants, especially first-line drugs such as SSRIs and SNRIs. Availability trends observed in the procurement data suggest a shift toward newer-generation antidepressants, primarily first-line drugs, gradually replacing traditional TCAs, accompanied by a substitution of generic drugs for branded ones. Although regional disparities in affordability appeared to have narrowed over the study period, gaps in availability were still observed, especially in lower-tier health facilities and less-developed provinces.

These findings, based on observed procurement patterns, suggest several practical implications for health policy and planning. In particular, policy efforts may benefit from strengthening pharmaceutical supply chains, expanding the procurement of essential antidepressants in under-resourced areas, and supporting lower-tier facilities to maintain a consistent medicine supply. In addition, promoting the use of high-quality generic medicines and improving the equitable distribution of newer-generation antidepressants may help enhance patient access to treatment across regions. Integrating pharmaceutical reimbursement data in future research could further clarify the economic impact on patients and help evaluate how policy measures influence equitable access to antidepressant treatment.

## Supplementary Material

Supplementary.docx

## Data Availability

Data cannot be shared publicly because we are not authorized by the owner (the Chinese Pharmaceutical Association). Data are available from the CPA (contact via Yingfeng Xu, phone 8610-65662756) for researchers who meet the criteria for access to confidential data.
