# Supplementary material for: Comparison of affordability and availability of antidepressants in China from 2019 to 2022: evidence from national procurement database
Source: J Pharm Policy Pract. 2026 Oct 14;19(1):2719527. doi: 10.1080/20523211.2026.2719527 (PMC13670268; doi:10.1080/20523211.2026.2719527)
Supplement: Supplementary file 1 [file JPPP_A_2719527_SM7210.docx]

**Supplementary Table 1.** Distribution of Health Facilities by Province, Stratified by Level and Ownership

|  |  | Level | | | Ownership | |
| --- | --- | --- | --- | --- | --- | --- |
| Region | Province | Primary | Secondary | Tertiary | Private | Public |
| East | Beijing | 1104 | 273 | 92 | 253 | 1216 |
|  | Tianjin | 144 | 106 | 37 | 8 | 279 |
|  | Hebei | 796 | 600 | 128 | 98 | 1426 |
|  | Shanghai | 664 | 192 | 61 | 223 | 694 |
|  | Jiangsu | 550 | 232 | 164 | 70 | 876 |
|  | Zhejiang | 381 | 258 | 160 | 42 | 757 |
|  | Fujian | 861 | 322 | 103 | 72 | 1214 |
|  | Shandong | 1117 | 438 | 192 | 131 | 1616 |
|  | Guangdong | 575 | 410 | 235 | 82 | 1138 |
|  | Hainan | 54 | 34 | 25 | 2 | 111 |
| Middle | Shanxi | 136 | 321 | 68 | 119 | 406 |
|  | Anhui | 161 | 170 | 96 | 7 | 420 |
|  | Jiangxi | 450 | 238 | 77 | 10 | 755 |
|  | Henan | 766 | 498 | 155 | 200 | 1219 |
|  | Hubei | 268 | 222 | 113 | 12 | 591 |
|  | Hunan | 235 | 278 | 98 | 62 | 549 |
| West | Inner Mongolia | 248 | 186 | 59 | 4 | 489 |
|  | Guangxi | 981 | 266 | 92 | 8 | 1331 |
|  | Chongqing | 149 | 131 | 41 | 17 | 304 |
|  | Sichuan | 792 | 261 | 257 | 16 | 1294 |
|  | Guizhou | 75 | 138 | 49 | 12 | 250 |
|  | Yunnan | 983 | 334 | 80 | 45 | 1352 |
|  | Tibet | 22 | 8 | 7 |  | 37 |
|  | Shaanxi | 202 | 276 | 80 | 13 | 545 |
|  | Gansu | 93 | 162 | 46 | 9 | 292 |
|  | Qinghai | 36 | 39 | 26 | 2 | 99 |
|  | Ningxia | 20 | 43 | 20 | 4 | 79 |
|  | Xinjiang | 82 | 176 | 49 | 20 | 287 |
| Northeast | Liaoning | 228 | 159 | 127 | 21 | 493 |
|  | Jilin | 37 | 66 | 42 | 13 | 132 |
|  | Heilongjiang | 59 | 143 | 63 | 12 | 253 |

**Supplementary Table 2.** Profile of Antidepressants: Key Characteristics

| Drug | Batch of NVBP | Originator | Mechanism | NEML | G | WHO EML |
| --- | --- | --- | --- | --- | --- | --- |
| Agomelatine |  | Les Laboratoires Servier | MRA |  |  |  |
| Amitriptyline |  | AstraZeneca | TCA | Y | Y | Y |
| Citalopram | 3 | Lundbeck | SSRI |  |  | A |
| Clomipramine |  | Novartis | TCA | Y |  |  |
| Doxepin |  |  | TCA | Y |  |  |
| Duloxetine | 4 | Lilly | SNRI |  |  |  |
| Escitalopram | 1 | Lundbeck | SSRI | Y |  | A |
| Fluoxetine | 3 | Patheon France, Lilly | SSRI | Y |  | Y |
| Flupentixol-Melitracen | 7 | Lundbeck | Combination |  |  |  |
| Fluvoxamine |  | Solvay, Abbott | SSRI |  |  | A |
| Maprotiline |  | Novartis | TCA |  |  |  |
| Mianserin |  | Merck Sharp & Dohme | TCA |  |  |  |
| Milnacipran |  | Pierre Fabre | SNRI |  |  |  |
| Mirtazapine |  | Organon, Merck Sharp & Dohme | NaSSA |  |  |  |
| Paroxetine | 1 | GlaxoSmithKline, Bora Pharmaceuticals | SSRI | Y |  | A |
| Reboxetine |  | Axsome Therapeutics | NARI |  |  |  |
| Sertraline | 3 | Pfizer | SSRI |  |  | A |
| Trazodone |  | Angelini | SARI |  |  |  |
| Venlafaxine | 5 | Wyeth, Pfizer | SNRI | Y |  |  |

Notes: SSRI, selective serotonin reuptake inhibitor; SNRI, serotonin noradrenaline reuptake inhibitor; TCA, tricyclic antidepressant; MRA, melatonin receptor agonist; NaSSA, noradrenergic and specific serotonergic antidepressant; NARI, noradrenaline reuptake inhibitor; SARI, serotonin receptor antagonist/reuptake inhibitor.

**Supplementary Table 3.** Number of Manufacturers for Each Drug, Distinguishing Between Original and Generic

|  | 2019 | | 2020 | | 2021 | | 2022 | |
| --- | --- | --- | --- | --- | --- | --- | --- | --- |
| Drug | Generic | OB | Generic | OB | Generic | OB | Generic | OB |
| Mianserin | 1 |  | 1 |  | 1 |  | 1 |  |
| Milnacipran | 1 |  | 1 |  | 1 |  | 1 |  |
| Reboxetine | 1 |  | 1 |  | 1 |  | 1 |  |
| Amitriptyline | 2 |  | 2 |  | 2 |  | 2 |  |
| Maprotiline | 3 |  | 3 |  | 3 |  | 3 |  |
| Clomipramine | 4 |  | 4 |  | 4 |  | 4 |  |
| Doxepin | 7 |  | 7 |  | 7 |  | 7 |  |
| Trazodone | 4 |  | 5 |  | 5 | Y | 5 | Y |
| Agomelatine | 1 |  | 1 | Y | 1 | Y | 1 | Y |
| Citalopram | 7 | Y | 7 | Y | 7 | Y | 6 | Y |
| Duloxetine | 5 | Y | 6 | Y | 8 | Y | 8 | Y |
| Escitalopram | 7 | Y | 7 | Y | 9 | Y | 13 | Y |
| Fluoxetine | 6 | Y | 6 | Y | 6 | Y | 6 | Y |
| Flupentixol-Melitracen | 4 | Y | 4 | Y | 4 | Y | 5 | Y |
| Fluvoxamine | 1 | Y | 1 | Y | 1 | Y | 1 | Y |
| Mirtazapine | 4 | Y | 4 | Y | 4 | Y | 4 | Y |
| Paroxetine | 4 | Y | 5 | Y | 5 | Y | 5 | Y |
| Sertraline | 16 | Y | 14 | Y | 13 | Y | 12 | Y |
| Venlafaxine | 11 | Y | 11 | Y | 13 | Y | 15 | Y |

Notes: OB, originator brand.

**Supplementary Table 4.** Regional Availability of Antidepressants, Grouped According to Mechanisms of Action and Pricing

|  | Availability | | | | | | MPR | | | | | |
| --- | --- | --- | --- | --- | --- | --- | --- | --- | --- | --- | --- | --- |
|  | LPG | | | OB | | | LPG | | | OB | | |
|  | 2019 | 2022 | Change | 2019 | 2022 | Change | 2019 | 2022 | Change | 2019 | 2022 | Change |
| ***East*** |  |  |  |  |  |  |  |  |  |  |  |  |
| First-line (NVBP) | 55.1% | 59.4% | 7.9% | 34.8% | 27.2% | -21.9% | 1.80 | 0.82 | -54.6% | 3.06 | 2.83 | -7.4% |
| First-line (Non-NVBP) | 12.0% | 16.2% | 34.2% | 4.9% | 4.6% | -7.7% | 2.99 | 2.60 | -13.0% | 1.73 | 2.42 | 39.8% |
| Second-line (Non-NVBP) | 35.7% | 38.8% | 8.7% | 4.8% | 6.9% | 43.5% | 1.34 | 1.83 | 36.9% | 4.47 | 4.49 | 0.4% |
| ***Middle*** |  |  |  |  |  |  |  |  |  |  |  |  |
| First-line (NVBP) | 51.1% | 61.6% | 20.6% | 25.2% | 15.5% | -38.7% | 1.79 | 0.79 | -56.0% | 3.26 | 2.48 | -23.9% |
| First-line (Non-NVBP) | 13.4% | 16.6% | 24.1% | 0.7% | 1.8% | 167.5% | 2.18 | 2.04 | -6.7% | 1.85 | 3.23 | 74.7% |
| Second-line (Non-NVBP) | 58.9% | 47.3% | -19.7% | 2.6% | 3.9% | 47.5% | 1.20 | 1.78 | 48.8% | 4.73 | 4.97 | 5.0% |
| ***West*** |  |  |  |  |  |  |  |  |  |  |  |  |
| First-line (NVBP) | 60.1% | 68.2% | 13.4% | 31.8% | 18.8% | -40.8% | 1.91 | 0.52 | -72.9% | 2.44 | 1.73 | -29.1% |
| First-line (Non-NVBP) | 13.6% | 14.2% | 4.1% | 1.2% | 10.6% | 762.6% | 2.79 | 1.94 | -30.5% | 1.89 | 2.15 | 13.7% |
| Second-line (Non-NVBP) | 51.4% | 33.4% | -35.0% | 2.9% | 3.0% | 0.8% | 1.43 | 1.77 | 23.8% | 4.57 | 4.35 | -4.9% |
| ***Northeast*** |  |  |  |  |  |  |  |  |  |  |  |  |
| First-line (NVBP) | 69.7% | 70.0% | 0.5% | 30.6% | 35.9% | 17.4% | 1.84 | 0.93 | -49.5% | 3.43 | 2.63 | -23.2% |
| First-line (Non-NVBP) | 12.2% | 18.9% | 54.8% | 1.4% | 2.8% | 105.4% | 2.63 | 2.54 | -3.5% | 1.83 | 2.04 | 11.6% |
| Second-line (Non-NVBP) | 28.6% | 35.4% | 23.8% | 1.5% | 5.4% | 270.3% | 2.16 | 2.44 | 13.2% | 4.50 | 4.45 | -1.0% |

Notes: Affordability < 1 indicates that the cost of a 30-day treatment is less than one day of minimum wage. Availability is operationalized as the percentage of health facilities that supplied specific antidepressants during the survey year. MPR, median price ratio against internationl reference unit price; LPG, lowest-priced generics; OB, originator brand; NVBP, National Volume-Based Procurement.

**Supplementary Table 5**. Availability of Antidepressants, Stratified by Health Facility Levels and Ownership Types

|  | Availability | | | | | | MPR | | | | | |
| --- | --- | --- | --- | --- | --- | --- | --- | --- | --- | --- | --- | --- |
|  | LPG | | | OB | | | LPG | | | OB | | |
|  | 2019 | 2022 | Change | 2019 | 2022 | Change | 2019 | 2022 | Change | 2019 | 2022 | Change |
| ***Primary (Pub.)*** |  |  |  |  |  |  |  |  |  |  |  |  |
| First-line (NVBP) | 45.5% | 50.3% | 10.6% | 18.3% | 15.4% | -15.6% | 1.65 | 0.68 | -58.8% | 1.55 | 1.62 | 4.3% |
| First-line (Non-NVBP) | 5.8% | 8.3% | 44.5% | 2.9% | 4.8% | 67.2% | 2.58 | 1.98 | -23.3% | 0.66 | 1.90 | 188.4% |
| Second-line (Non-NVBP) | 31.2% | 26.3% | -15.7% | 1.6% | 2.1% | 32.1% | 0.95 | 1.46 | 53.6% | 4.53 | 4.34 | -4.2% |
| ***Primary (Pri.)*** |  |  |  |  |  |  |  |  |  |  |  |  |
| First-line (NVBP) | 38.7% | 55.8% | 44.1% | 34.2% | 20.8% | -39.1% | 1.55 | 0.80 | -48.4% | 3.20 | 2.88 | -9.9% |
| First-line (Non-NVBP) | 3.3% | 5.3% | 60.7% | 1.8% | 2.1% | 16.0% | 2.30 | 2.31 | 0.4% | 1.83 | 2.38 | 30.6% |
| Second-line (Non-NVBP) | 20.0% | 22.3% | 11.2% | 3.7% | 3.4% | -8.4% | 1.01 | 1.83 | 80.7% | 4.60 | 4.49 | -2.4% |
| ***Secondary (Pub.)*** |  |  |  |  |  |  |  |  |  |  |  |  |
| First-line (NVBP) | 64.4% | 73.9% | 14.7% | 40.0% | 27.4% | -31.5% | 1.74 | 0.63 | -63.6% | 2.17 | 2.71 | 24.4% |
| First-line (Non-NVBP) | 15.8% | 20.6% | 30.5% | 2.8% | 5.8% | 106.6% | 2.45 | 2.15 | -12.3% | 1.76 | 2.26 | 28.1% |
| Second-line (Non-NVBP) | 50.8% | 51.2% | 0.9% | 3.9% | 6.0% | 53.4% | 1.21 | 1.79 | 47.8% | 4.56 | 4.64 | 1.7% |
| ***Secondary (Pri.)*** |  |  |  |  |  |  |  |  |  |  |  |  |
| First-line (NVBP) | 61.1% | 75.0% | 22.9% | 45.0% | 18.7% | -58.4% | 2.05 | 0.74 | -64.1% | 3.15 | 2.92 | -7.3% |
| First-line (Non-NVBP) | 6.7% | 8.9% | 32.8% |  | 2.7% |  | 1.12 | 1.71 | 52.7% |  | 2.35 |  |
| Second-line (Non-NVBP) | 45.0% | 25.8% | -42.5% | 3.4% | 2.1% | -36.3% | 1.06 | 1.50 | 41.8% | 4.69 | 4.36 | -7.0% |
| ***Tertiary (Pub.)*** |  |  |  |  |  |  |  |  |  |  |  |  |
| First-line (NVBP) | 80.6% | 88.2% | 9.3% | 62.1% | 44.7% | -28.0% | 1.87 | 0.76 | -59.2% | 3.23 | 2.70 | -16.2% |
| First-line (Non-NVBP) | 32.3% | 40.8% | 26.5% | 6.2% | 11.3% | 81.8% | 2.86 | 2.48 | -13.4% | 1.81 | 2.48 | 36.9% |
| Second-line (Non-NVBP) | 69.0% | 70.6% | 2.2% | 11.4% | 16.5% | 44.3% | 1.54 | 1.94 | 26.4% | 4.48 | 4.51 | 0.6% |
| ***Tertiary (Pri.)*** |  |  |  |  |  |  |  |  |  |  |  |  |
| First-line (NVBP) | 64.3% | 88.6% | 37.9% | 39.3% | 11.4% | -71.1% | 1.78 | 0.61 | -65.7% | 2.79 | 2.83 | 1.4% |
| First-line (Non-NVBP) | 28.6% | 8.3% | -70.8% | 3.6% | 2.3% | -36.4% | 4.04 | 2.81 | -30.5% | 1.83 | 1.87 | 2.3% |
| Second-line (Non-NVBP) | 53.6% | 16.7% | -68.9% | 10.7% | 3.0% | -71.7% | 1.73 | 1.58 | -8.9% | 4.53 | 4.27 | -5.8% |

Notes: Affordability < 1 indicates that the cost of a 30-day treatment is less than one day of minimum wage. Availability is operationalized as the percentage of health facilities that supplied specific antidepressants during the survey year. MPR, median price ratio against internationl reference unit price; LPG, lowest-priced generics; OB, originator brand; NVBP, National Volume-Based Procurement; Pub., public; Pri., private.
